# Serum and urine FGF23 and IGFBP-7 for the prediction of acute kidney injury in critically ill children

**DOI:** 10.1186/s12887-018-1175-y

**Published:** 2018-06-15

**Authors:** Zhenjiang Bai, Fang Fang, Zhong Xu, Chunjiu Lu, Xueqin Wang, Jiao Chen, Jian Pan, Jian Wang, Yanhong Li

**Affiliations:** 1 Pediatric Intensive Care Unit, Children’s Hospital of Soochow University, Suzhou, JiangSu province China; 2 Institute of Pediatric Research, Children’s Hospital of Soochow University, Suzhou, JiangSu province China; 3 Department of nephrology, Institute of pediatric research, Children’s Hospital of Soochow University, Suzhou, JiangSu province China

**Keywords:** Acute kidney injury, Critically ill children, Cystatin C, Fibroblast growth factor 23, Insulin-like growth factor binding protein 7, Pediatric risk of mortality III score

## Abstract

**Background:**

Fibroblast growth factor 23 (FGF23) and insulin-like growth factor binding protein 7 (IGFBP-7) are suggested to be biomarkers for predicting acute kidney injury (AKI). We compared them with proposed AKI biomarker of cystatin C (CysC), and aimed (1) to examine whether concentrations of these biomarkers vary with age, body weight, illness severity assessed by pediatric risk of mortality III score, and kidney function assessed by estimated glomerular filtration rate (eGFR), (2) to determine the association between these biomarkers and AKI, and (3) to evaluate whether these biomarkers could serve as early independent predictors of AKI in critically ill children.

**Methods:**

This prospective single center study included 144 critically ill patients admitted to the pediatric intensive care unit (PICU) regardless of diagnosis. Serum and spot urine samples were collected during the first 24 h after PICU admission. AKI was diagnosed based on the AKI network (AKIN) criteria.

**Results:**

Twenty-one patients developed AKI within 120 h of sample collection, including 11 with severe AKI defined as AKIN stages 2 and 3. Serum FGF23 levels were independently associated with eGFR after adjustment in a multivariate linear analysis (*P* < 0.001). Urinary IGFBP-7 (Adjusted OR = 2.94 per 1000 ng/mg increase, *P* = 0.035), serum CysC (Adjusted OR = 5.28, *P* = 0.005), and urinary CysC (Adjusted OR = 1.13 per 1000 ng/mg increase, *P* = 0.022) remained significantly associated with severe AKI after adjustment for body weight and illness severity, respectively. Urinary IGFBP-7 level was predictive of severe AKI and achieved the AUC of 0.79 (*P* = 0.001), but was not better than serum (AUC = 0.89, *P* < 0.001) and urinary (AUC = 0.88, *P* < 0.001) CysC in predicting severe AKI.

**Conclusions:**

Serum FGF23 levels were inversely related to measures of eGFR. In contrast to serum and urinary FGF23 which are not associated with AKI in a general and heterogeneous PICU population, an increased urinary IGFBP-7 level was independently associated with the increased risk of severe AKI diagnosed within the next 5 days after sampling, but not superior to serum or urinary CysC in predicting severe AKI in critically ill children.

## Background

Critically ill children are at a high risk of developing acute kidney injury (AKI), which is an independent risk factor associated with high mortality and morbidity [1–4]. Research in AKI has focused on identifying biomarkers for early diagnosis, which is crucial to initiate effective therapies [5–10]. Although potential biomarkers for predicting AKI have been identified during the last decade, strong evidence is still lacking to confirm that early biomarkers of AKI have beneficial effects on the clinical outcomes in a general intensive care unit (ICU) population, which leads to attempts to identify novel biomarkers that can predict the development of AKI at an earlier stage [5, 7, 11, 12]. Two of the emerging biomarkers of AKI are fibroblast growth factor 23 (FGF23) [13–19] and insulin-like growth factor binding protein 7 (IGFBP-7) [20–24].

FGF23, a circulating 26-kDa peptide produced by osteocytes, plays an important role in regulating phosphate and vitamin D homeostasis as a phosphate-regulating hormone [13]. Although it has been studied less extensively in AKI, a number of previous studies revealed that plasma FGF23 levels rise rapidly during AKI, suggesting that plasma FGF23 has the potential to diagnose AKI [15–19]. In adult patients undergoing cardiac surgery [18] or in children undergoing cardiopulmonary bypass [19], plasma FGF23 was significantly higher and independently associated with adverse outcomes [18]. So far, two studies of FGF23 with small sample size have been carried out in adult ICU patients [14, 15]. Elevated level of FGF23 was reported in a cohort of 12 ICU patients with AKI compared with 8 control ICU patients without AKI [14]. Subsequently, a prospective observational study of 60 hospitalized adult patients, including 27 from ICU, showed that FGF23 level is elevated and associated with greater risk of death or need for renal replacement therapy [15]. Analysis of larger cohorts is necessary to see if these findings can be replicated in general ICU patients, and whether these findings can apply to critically ill children remains unclear.

IGFBP-7, also known as IGFBP-related protein 1 (IGFBP-rP1), is an additional member of the IGFBP family and involved with the phenomenon of G1 cell-cycle arrest [24]. Renal tubular cells can enter a short period of G1 cell-cycle arrest during the very early phases of cell injury, representing an early response to renal injury [25]. Indeed, urinary IGFBP-7 was identified by proteomics as an early prognostic marker of AKI severity [20]. IGFBP-7 and tissue inhibitor of metalloproteinases-2 (TIMP-2) were further validated in a large multicenter of ICU patients as a predictor of AKI defined by risk, injury, failure, loss, end-stage renal disease (RIFLE) criteria, suggesting that the urinary concentration of IGFBP7 multiplied by TIMP-2 is a novel prognostic urinary biomarker of AKI [23, 24]. However, whether IGFBP-7 alone is a new candidate predictive biomarker of AKI remains to be validated. Serum IGFBP-7 was reported to be associated with insulin resistance and diabetes [26] that may have direct renal effects, resulting in glomerular hyperfiltration and renal damage [27]. However, whether serum IGFBP-7 correlates with renal function, and whether there is a relationship between the serum IGFBP-7 concentration and urinary IGFBP-7 excretion remain elucidated.

In the present study, we assessed concentrations of both FGF23 and IGFBP-7 in serum and urine, and compared them with proposed biomarkers of AKI, serum and urinary cystatin C (CysC). We aimed (1) to examine whether concentrations of these biomarkers vary with age, body weight, and illness severity as assessed by the pediatric risk of mortality III (PRISM III) score, as well as with kidney function as assessed by estimated glomerular filtration rate (eGFR) in critically ill children, (2) to determine the association between these biomarkers and AKI, and (3) to evaluate whether serum and urinary FGF23 and IGFBP-7 could serve as early predictors of AKI, independently of potential confounders, in critically ill children.

## Methods

### Cohorts, setting, and data collection

All patients who were admitted to the pediatric ICU (PICU) regardless of diagnosis in the university-affiliated tertiary children hospital from May to August 2012 were considered for inclusion in the prospective study. The criteria for PICU admission in our hospital were adopted from guidelines for developing admission and discharge policies for the PICU, as described previously [28, 29], including both medical and surgical patients and age between 1 month and 16 years. The exclusion criteria were the presence of congenital abnormality of the kidney, discharge from PICU before sampling, and unexpected discharge from the PICU or withdrawal of therapy. The Institutional Review Board of the Children’s Hospital of Soochow University approved the study. Informed parental written consent was obtained at enrollment of each patient, and all clinical investigations were conducted according to the principles expressed in the Declaration of Helsinki.

### Assessment of illness severity

The PRISM III score, based on age-related physiological parameters collected in the first 24 h after PICU admission, was used as a measure to assess illness severity of critically ill children [30].

### Diagnosis of AKI

The diagnosis of AKI developed within 120 h of sample collection was based on the serum creatinine (Cr) level defined by the AKI network (AKIN) criteria [1, 31] without urine output criteria. For patients with elevated serum Cr ≥ 106.1 μmol/L at PICU admission, the lowest Cr value during hospitalization was considered as the baseline Cr, in accordance with previous studies [32, 33]. Severity of AKI was characterized by the AKIN criteria. AKIN stage 1 was defined as mild AKI, and AKIN stages 2 and 3 were defined as severe AKI.

### Measurement of serum and urinary FGF23 and IGFBP-7

Non-fasting venous blood and spot urine were collected during the first 24 h after PICU admission and immediately aliquoted and stored at − 80 °C. Serum and urine were first centrifuged at 1500×g at 4 °C for 15 min and the supernatants were used for the measurement. The FGF23 level was quantified by the human enzyme-linked immunosorbent assay (ELISA) kit (SEA746Hu, Cloud-Clone Corp, USA), according to the manufacturer’s protocol. The minimum detectable level of FGF23 was < 6.7 pg/mL, and the coefficient of variation of intra-assay and inter-assay were less than 10 and 12% respectively, corresponding to that reported by the manufacturer. The FGF23 levels were detectable in all serum samples and in 118 (81.9%) urinary samples. For those samples with undetectable FGF23 levels (18.1%), the FGF23 value was assumed to have a concentration at 6.7 pg/mL equivalent to the detection limit of the assay to facilitate the calculation for urinary FGF23/urinary Cr ratios.

The human IGFBP-rp1/IGFBP-7 ELISA kit (DY1334–05, R&D Systems, USA) was used for the measurement. The samples were diluted 20-fold to 100-fold in Reagent Diluent to ensure that the enzymatic reaction was maintained within the linear range. The coefficient of variation of intra-assay and inter-assay were less than 10%. The level of IGFBP-7 was detectable in all samples.

### Measurement of serum and urinary CysC and Cr

The levels of CysC and Cr from the aliquoted samples were measured on an automatic biochemical analyzer (Hitachi 7600, Japan), as described previously [6]. The CysC level was measured using latex enhanced immunoturbidimetry assay, and the detection limit for CysC was 0.01 mg/L. The coefficient of variation of intra-assay and inter-assay were ≤ 10%. The CysC levels were detectable in all serum samples and in 131 (91.0%) urinary samples. Urinary CysC values for those with undetectable CysC levels were assumed to have the concentration at 0.01 mg/L equivalent to the detection limit of the assay for calculation of the urinary CysC/urinary Cr ratio. The serum and urinary Cr levels were measured automatically using the sarcosine oxidase method on the automatic biochemical analyzer.

### Estimated glomerular filtration rate

Estimated GFR was calculated according to the following formula published by Bouvet et al. [34]: eGFR (ml/min) = 63.2× [1.2/serum CysC (mg/L)]^0.56^x [1.09/serum Cr (mg/dL)]^0.35^x [weight (kg)/45]^0.3^x [age (years)/14]^0.4^. The results of Cr and CysC were obtained from the aliquoted serum samples.

### Statistical analysis

Data analyses were performed using SPSS statistical software. We first checked assumptions of normality and homogeneity of variance. The Mann-Whitney U test was used to analyze differences between two groups, and the Kruskal-Wallis H test was used to analyze differences among three groups. The chi-square test or Fisher’s exact test were used to compare differences in categorical variables among groups. Spearman’s analysis was performed to examine correlations. Univariate and multivariate linear analyses were used to analyze the association of variables with eGFR. The data for continuous variables were log-transformed to meet the assumptions of homogeneity of variances. Univariate and multivariate logistic regression analyses were used to calculate odds ratio (OR) to assess the association of biomarkers with AKI, and to identify independent variables associated with AKI. Model fit was assessed by the Hosmer-Lemeshow goodness-of-fit test with *P* > 0.05, suggesting the absence of a biased fit. The area under-the-receiver-operating-characteristic curve (AUC) was calculated to assess the predictive strength, and the nonparametric method of Delong was performed to compare differences between AUCs. Optimal cut-off points to maximize both sensitivity and specificity were determined using Sigma Plot 10.0 software.

## Results

### Patient characteristics

The study involved 144 critically ill children. Of a total of 179 children were admitted to the PICU during the study period, 35 were excluded: 2 died and 5 were discharged from PICU before sampling, 3 had withdrawal of therapy, and 25 had a failure in collecting blood and urine samples during the first 24 h after PICU admission. The leading cause of PICU admission in the cohort was neurologic diseases (33.3%), followed by respiratory diseases (30.6%). Twenty-four (16.7%) patients were diagnosed with sepsis.

Of the 144 patients, 21 (14.6%) developed AKI within 120 h of sample collection. Ten patients fulfilled the AKIN criteria stage 1 defined as mild AKI: 5 on the first, 3 on the second, 1 on the third, and 1 on the fifth day after PICU admission. Eleven patients fulfilled the criteria of AKIN stages 2 and 3 defined as severe AKI, including 6 patients developed AKIN stage 2: 5 on the first and 1 on the third day after admission; and 5 patients developed AKIN stage 3: 2 on the first, 2 on the second, and 1 on the fourth day after admission.

A comparison of the demographic and clinical characteristics and outcomes among patients with non-AKI, mild AKI, and severe AKI is displayed in Table 1.

**Table 1:** Demographic and clinical characteristics grouped according to AKI status

Variable	Non-AKI	Mild AKI	Severe AKI	*P*
	(*n* = 123)	(*n* = 10)	(*n* = 11)	
Age, months	12 [4–48]	30.5 [11.25–98]	59 [4–98]	0.049^&^
Body weight, kg	10 [6.5–14]	14 [8.75–26.25]	20 [6.5–30]*	0.024^&^
Male, n	70 (56.9)	5 (50.0)	7 (63.6)	0.819
PRISM III score	3 [0.25–6.75]	7.5 [4.25–10.5]*	17 [8–20]*^#^	< 0.001
Arterial pH^a^	7.409 [7.363–7.468]	7.461 [7.392–7.481]	7.400 [7.203–7.497]	0.297
Blood bicarbonate^a^, mmol/L	20.0 [17.6–22.2]	17.1 [15.5–20.0]*	17.1 [8.1–19.6]*	0.020^φ^
Serum albumin^a^, g/L	41.7 [38.5–44.4]	40.2 [34.9–46.9]	35.3 [26.7–43.8]*	0.026^φ^
Serum creatinine^a^, μmol/L	24.6 [19.5–31.8]	44.3 [26.9–72.1]*	86.4 [77.3–140.0]*^#^	< 0.001^φ^
Blood urea nitrogen^a^, μmol/L	3.30 [2.54–4.40]	6.34 [3.41–8.53]*	7.00 [5.84–13.44]*	< 0.001^φ^
Serum sodium^a^, μmol/L	134.6 [132.3–136.6]	135.8 [133.2–140.3]	132.8 [130.3–133.7]*^#^	0.008^ζ^
Serum potassium^a^, μmol/L	4.02 [3.57–4.56]	4.31 [3.77–4.47]	4.32 [3.83–5.60]	0.157
MODS^b^, n	3 (2.4)	2 (20.0)*	6 (54.5)^*^	< 0.001^φ^
Shock/DIC^b^, n	11 (8.9)	2 (20.0)	5 (45.5)^*^	< 0.001^ζ^
MV^c^, n	45 (36.6)	6 (60.0)	10 (90.9)^*^	0.001^ζ^
Duration of MV^c^, hours	0 [0–44]	35 [0–123.5]	115 [12–134]^*^	0.001^ζ^
Prolonged MV (> 48 h)^c^, n	26 (21.1)	4 (40.0)	8 (72.7)^*^	0.002^φ^
Antibiotics^c^, n	116 (94.3)	10 (100)	11 (100)	0.322
Inotrope^c^, n	23 (18.7)	1 (10.0)	8 (72.7)*^#^	0.001^φ^
Furosemide^c^, n	31 (25.2)	3 (30.0)	11 (100)*^#^	0.032^φ^
Steroids^c^, n	45 (36.6)	3 (30.0)	5 (45.5)	0.757
PICU LOS, hours	66 [36–141]	77.5 [38.25–256]	152 [118–181]*	0.032^ζ^
Death, n	5 (4.1)	1 (10.0)	2 (18.2)	0.093

Values are median [interquartile range]. Numbers in parentheses denote percentages

AKI network stage 1 was defined as mild AKI, and AKIN stages 2 and 3 were defined as severe AKI. *AKI* acute kidney injury, *DIC* disseminated intravascular coagulation, *LOS* length of stay, *MODS* multiple organ dysfunction syndrome, *MV* mechanical ventilation, *PICU* pediatric intensive care unit, *PRISM III* pediatric risk of mortality III

^a^The first available laboratory results during the first 24 h after PICU admission. ^b^Developed during PICU stay. ^c^Administration during PICU stay

*P < 0.05, compared with non-AKI; ^#^*P* < 0.05, compared with mild AKI. ^&^*P* > 0.05, after adjustment for PRISM III score. ^ζ^*P* > 0.05, ^φ^*P* < 0.05, after adjustment for body weight and PRISM III score

### Correlation of serum and urinary biomarkers with age, body weight, gender, sepsis, and illness severity

Spearman’s correlation analyses of biomarkers with age, body weight, gender, sepsis, and PRISM III score are displayed in Table 2. Multivariate linear regression analyses, including variables of age, body weight, gender, sepsis, and PRISM III score, were further performed. Serum levels of FGF23 (*P* = 0.010) and CysC (*P* = 0.003) remained independently associated with age. In addition, when we grouped the patients into two age categories: ≤3 years (*n* = 102) and > 3 years (*n* = 42), the negative correlation between age and serum FGF23 levels was only significant in patients aged ≤3 years (*r* = − 0.590, *P* < 0.001), but not in patients aged > 3 years (*r* = 0.064, *P* = 0.682). Moreover, the correlation of sepsis with serum FGF23 (*P* = 0.068), urinary IGFBP-7 (*P* = 0.350), and urinary CysC (*P* = 0.391), however, did not remain significant after adjustment for age, body weight and illness severity in a multivariate analysis.

**Table 2:** Correlation of biomarkers with age, body weight, gender, sepsis, and illness severity

Variable	Statistics	sFGF23 pg/mL	sIGFBP-7 ng/mL	sCysC mg/L	uFGF23 pg/mg uCr	uIGFBP-7 ng/mg uCr	uCysC ng/mg uCr
Age, months	r	−0.608	− 0.274	− 0.369	− 0.209	0.049	− 0.114
	*P*	< 0.001*	0.001	< 0.001*	0.012	0.556	0.175
Body weight, kg	r	−0.598	− 0.253	− 0.346	−0.233	0.066	−0.102
	*P*	< 0.001	0.002	< 0.001	0.005	0.433	0.224
Gender	Z	−0.051	−0.682	−0.077	−1.271	− 0.020	−0.444
	*P*	0.959	0.495	0.939	0.204	0.984	0.657
Sepsis	Z	−2.144	−1.812	−.901	− 1.614	−2.037	−2.589
	*P*	0.032	0.070	0.368	0.107	0.042	0.010
PRISM III score	r	−0.002	0.093	0.084	0.054	0.327	0.253
	*P*	0.981	0.269	0.317	0.524	< 0.001*	0.002*

*PRISM III* pediatric risk of mortality III, r = Spearman’s correlation coefficient; Z: The Mann-Whitney U test

*P < 0.05, multivariate linear regression analysis, including variables of age, body weight, gender, and PRISM III score. Continuous variables were log-transformed in multivariate analysis

### Association of serum and urinary biomarkers with eGFR

Univariate and multivariate linear analyses were used to analyze the association of biomarkers with kidney function as assessed by eGFR. Serum levels of FGF23 (*P* < 0.001), IGFBP-7 (*P* = 0.003), and CysC (*P* < 0.001) and urinary levels of FGF23 (*P* = 0.001) and CysC (*P* = 0.022) were associated with eGFR in the univariate linear regression analysis in Table 3. To identify whether these biomarkers were independently associated with eGFR, the multivariate linear analysis was further conducted. The association of eGFR with serum FGF23 (*P* = 0.040) and urinary CysC (P = 0.001) remained significant in the multivariate analysis after adjustment for age and body weight, as shown in Table 3.

**Table 3:** Association of variables with eGFR

Variable	Univariate regression		Multivariate regression	
	B coefficient (SE)	*P*	B coefficient (SE)	*P*
Age, months	0.524 (0.025)	< 0.001		
Body weight, kg	1.129 (0.067)	< 0.001		
Gender	−0.063 (0.062)	0.317		
PRISM III score	0.000 (0.006)	0.959		
MV	−0.033 (0.063)	0.595		
Duration of MV, hours	0.000 (0.000)	0.302		
sFGF23, pg/mL	−0.842 (0.108)	< 0.001	−0.156 (0.075)^a^	0.040
sIGFBP-7, ng/mL	−0.657(0.214)	0.003	−0.111 (0.113)^a^	0.327
sCysC, mg/L	−1.062 (0.113)	< 0.001	−0.702 (0.048)^a^	< 0.001
uFGF23, pg/mg uCr	−0.169 (0.051)	0.001	−0.050 (0.027)^a^	0.061
uIGFBP-7, ng/mg uCr	−0.013 (0.065)	0.843		
uCysC, ng/mg uCr	−0.097 (0.042)	0.022	−0.067 (0.020)^a^	0.001

*eGFR* estimated glomerular filtration rate, *MV* mechanical ventilation, *PRISM III* pediatric risk of mortality III. eGFR was calculated based on age, body weight, and serum levels of creatinine and cystatin C

^a^After adjustment for age and body weight. All continuous variables were log-transformed

### Association of serum and urinary biomarkers with severe AKI

**Fig. 1:**
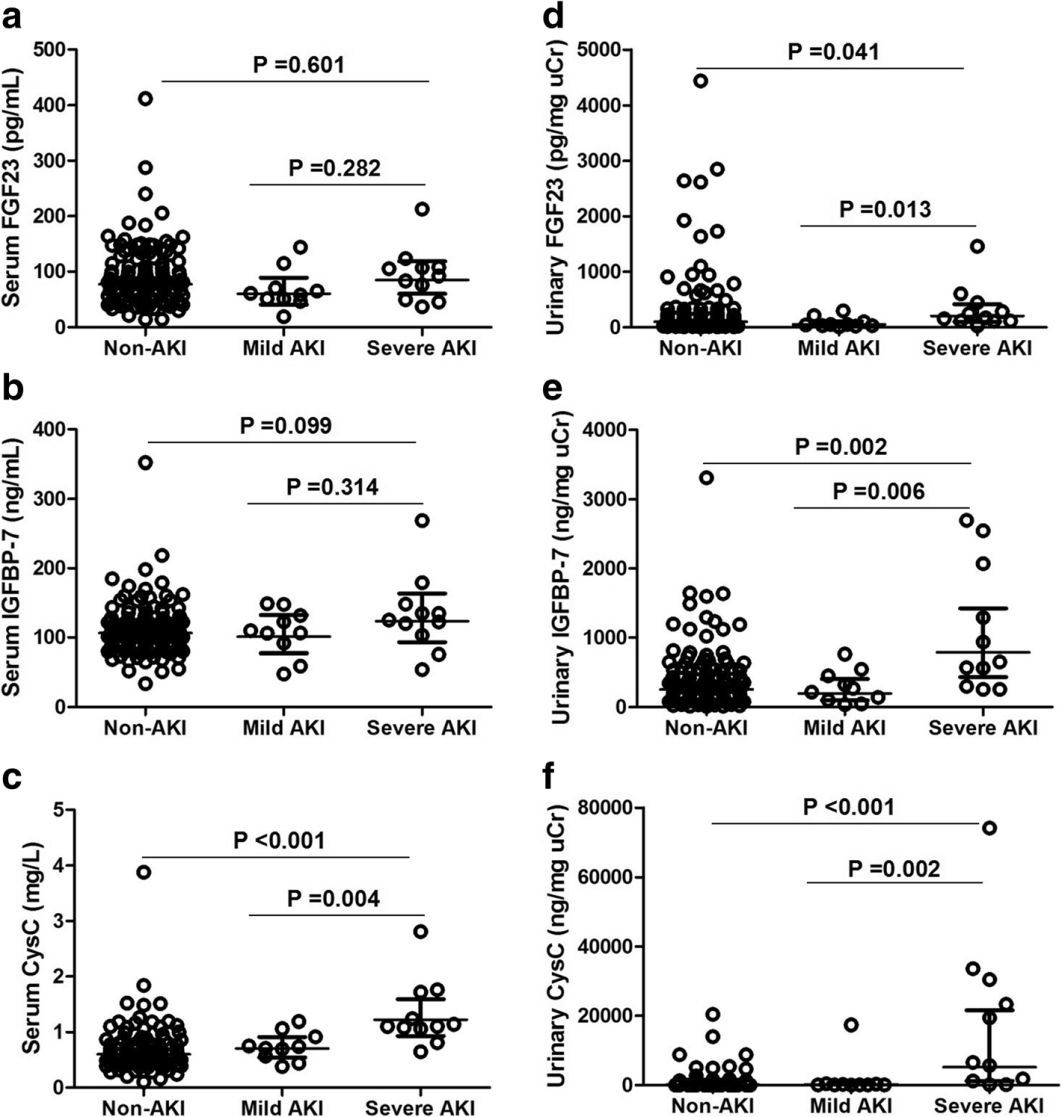
Comparison of the levels of biomarkers among critically ill children with non-AKI, mild AKI, and severe AKI. **a** serum level of FGF23, **b** serum level of IGFBP-7; **c** serum level of CysC, **d** urinary level of FGF23, **e** urinary level of IGFBP-7, **f** urinary level of CysC. AKI network stage 1 was defined as mild AKI. AKI network stages 2 and 3 were defined as severe AKI. Each circle represents an individual patient; the horizontal lines indicate geometric means with 95% confidence interval. Probability values: the Mann-Whitney U test. The *P* value for comparison between non-AKI (*n* = 123) and severe AKI (*n* = 11), and for comparison between mild (*n* = 10) and severe (*n* = 11) AKI

Comparisons of serum and urinary levels of FGF23, IGFBP-7, and CysC among patients with non-AKI, mild AKI, and severe AKI are shown in Table 4 and Fig. 1. Since there was no significant difference in serum and urinary levels of FGF23, IGFBP-7, and CysC between patients with mild AKI and without AKI (*P* > 0.05), univariate and multivariate logistic analyses were used to analyze the association of biomarkers with severe AKI in Table 5.

**Table 4:** Serum and urinary FGF23, IGFBP-7 and CysC levels grouped according to AKI status

Biomarker	Non-AKI	Mild AKI	Severe AKI	*P*
	(*n* = 123)	(*n* = 10)	(*n* = 11)	
sFGF23, pg/mL	79.33 [49.88–115.84]	59.97 [50.25–81.57]	92.33 [49.98–107.50]	0.372
sIGFBP-7, ng/mL	107.92 [87.47–125.02]	108.17 [83.65–135.71]	125.26 [103.07–148.35]	0.255
sCysC, mg/L	0.60 [0.47–0.78]	0.73 [0.54–0.96]	1.10 [1.06–1.72]*^#^	< 0.001
uFGF23, pg/mg uCr	74.40 [39.20–225.8]	47.14 [28.82–130.6]	172.93 [114.37–448.25]*^#^	0.033
uIGFBP-7, ng/mg uCr	291.57 [135.60–539.04]	244.33 [87.51–478.73]	653.50 [301.94–2072.06]*^#^	0.005
uCysC, ng/mg uCr	183.17 [94.62–494.96]	122.38 [80.27–332.97]	6559.79 [1224.42–30,414.64]*^#^	< 0.001

Values are median [interquartile range]

AKI network stage 1 was defined as mild AKI, and AKIN stages 2 and 3 were defined as severe AKI

*P < 0.05, compared with non-AKI; ^#^*P* < 0.05, compared with mild AKI

**Table 5:** Association of variables with severe AKI

Variable	OR	95% CI	*P*	AOR	95% CI	*P*
Age, months	1.01	1.00–1.03	0.026	1.01^d^	0.99–1.02	0.567
Body weight, kg	1.09	1.03–1.16	0.003	1.03^d^	0.96–1.12	0.428
Gender	0.74	0.21–2.65	0.642			
PRISM III score	1.36	1.18–1.55	< 0.001	1.32^e^	1.15–1.53	< 0.001
MV	16.08	2.00–129.36	0.009	5.03^f^	0.50–50.56	0.170
Duration of MV, hours	1.00	1.00–1.00	0.494			
Sepsis	3.23	0.87–12.05	0.081			
eGFR, mL/min	0.98	0.96–1.01	0.138			
sFGF23, pg/mL	1.00	0.99–1.01	0.730			
sIGFBP-7, ng/mL	1.01	0.99–1.02	0.096			
sCysC, mg/L	6.67	1.84–24.18	0.004	5.28^f, g^	1.64–16.99	0.005
uFGF23, pg/mg uCr	1.15^a^	0.47–2.82	0.761			
uIGFBP-7, ng/mg uCr	4.37^b^	1.82–10.49	0.001	2.94^b, f, g^	1.08–8.01	0.035
uCysC, ng/mg uCr	1.21^c^	1.10–1.34	< 0.001	1.13^c, f, g^	1.02–1.25	0.022

AKI, acute kidney injury; AOR, Adjusted OR; CI, confidence interval; eGFR, estimated glomerular filtration rate; MV, mechanical ventilation; OR, odds ratio; PRISM III, pediatric risk of mortality III

Severe AKI was defined as AKI network stages 2 and 3

^a^Odds ratio represents the increase in risk per 1000 pg/mg increase in uFGF23/uCr. ^b^Odds ratio represents the increase in risk per 1000 ng/mg increase in uIGFBP-7/uCr. ^c^Odds ratio represents the increase in risk per 1000 ng/mg increase in uCysC/uCr

^d^After adjustment for PRISM III score. ^e^After adjustment for age and body weight. ^f^After adjustment for body weight and PRISM III score. ^g^*P* < 0.05, after adjustment for body weight, sepsis, and PRISM III score

The association of serum CysC (*P* = 0.005), urinary IGFBP-7 (*P* = 0.035), and urinary CysC (*P* = 0.022) with severe AKI remained significant after controlling for body weight and illness severity as assessed by PRISM III score (Table 5).

### Ability of serum and urinary biomarkers to predict severe AKI

The predictive ability of serum and urinary CysC and urinary IGFBP-7 levels for severe AKI is shown in Table 6. Serum CysC displayed the highest AUC of 0.89 (*P* < 0.001), which was similar to the result obtained based on the PRISM III score (AUC = 0.92, *P* < 0.001), for predicting severe AKI in critically ill children, followed by urinary CysC (AUC = 0.88, *P* < 0.001).

**Table 6:** Predictive characteristics of biomarkers for severe AKI

Variable	AUC	95% CI	*P*	Optimal cut-off value	Sensitivity (%)	Specificity (%)
PRISM III score	0.92	0.84–0.99	< 0.001	7.5	90.9	77.4
sCysC, mg/L	0.89	0.82–0.97	< 0.001	0.81	90.9	78.2
uCysC, ng/mg uCr	0.88	0.76–0.99	< 0.001	1145.0	81.8	86.5
uIGFBP-7, ng/mg uCr	0.79	0.66–0.92	0.001	563.4	72.7	79.0
uIGFBP-7, combined with sCysC	0.89	0.79–0.99	< 0.001			
uIGFBP-7, combined with uCysC	0.88	0.79–0.98	< 0.001			
uIGFBP-7, combined with sCysC and uCysC	0.90	0.81–1.00	< 0.001			

Severe AKI was defined as AKI network stages 2 and 3

*AKI* acute kidney injury, *AUC* the area under the ROC curve, *CI* confidence interval, *PRISM III* pediatric risk of mortality III

**Fig. 2:**
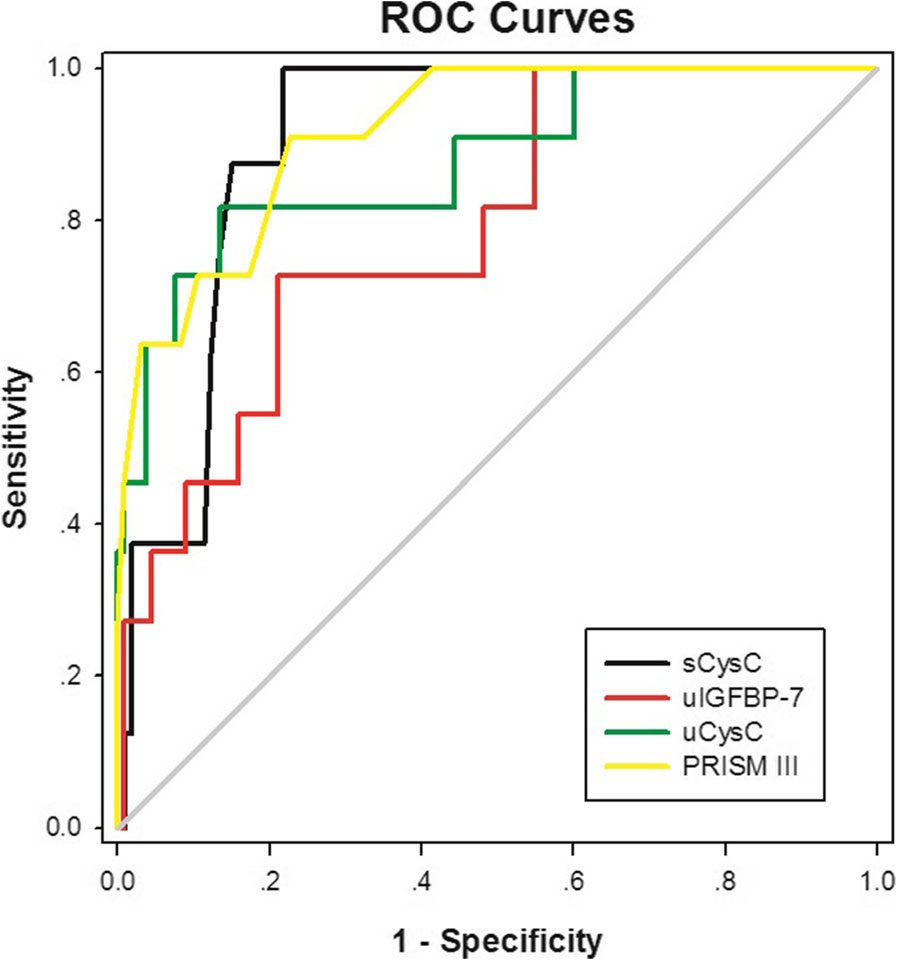
ROC curves for the ability of urinary IGFBP-7, serum and urinary cystatin C, and PRISM III score to predict severe AKI in critically ill children. AKI network stages 2 and 3 were defined as severe AKI. AKI, acute kidney injury; AUC, the area under the ROC curve; PRISM III, pediatric risk of mortality III; ROC, receiver operating characteristic. The *P* value for comparison between the AUCs of urinary IGFBP-7 and serum cystatin C was 0.103 and for comparison between the AUCs of urinary IGFBP-7 and urinary cystatin C was 0.225

Urinary IGFBP-7 level was predictive of severe AKI and achieved the AUC of 0.79 (*P* = 0.001), but was not better than serum CysC and urinary CysC, in predicting severe AKI. However, the difference between the two AUCs of either urinary IGFBP-7 (AUC = 0.79) and serum CysC (AUC = 0.89) (*P* = 0.103) or urinary IGFBP-7 and urinary CysC (AUC = 0.88) (*P* = 0.225) did not reach statistically significant. In addition, combining urinary IGFBP-7 with serum and urinary CysC improved the predictive performance, which was superior to urinary IGFBP-7 alone (*P* = 0.029), but not significantly better than serum CysC alone (*P* = 0.689). ROC curves for the ability of serum CysC, urinary IGFBP-7, urinary CysC, and PRISM III score to predict severe AKI in critically ill children are shown in Fig. 2.

## Discussion

Our results demonstrated that serum FGF23 level was inversely related to measures of eGFR, and an increased urinary level of IGFBP-7 was associated with the increased risk of severe AKI diagnosed within the next 5 days after sampling. However, urinary IGFBP-7 was not superior to serum or urinary CysC in predicting severe AKI in critically ill children.

Previous findings indicate that variables, such as age, gender, and illness severity, may interfere with CysC and other traditional renal biomarkers [6, 35]. We found that both serum CysC and FGF23 levels were independently associated with age. Serum CysC concentration has been reported to be gradually declined with increasing age in younger children less than 3 years old, which reflects renal maturation [35]. Similarly, the decreased serum FGF23 level with increasing age during the first 3 years of age as seen in the present study may also reflect renal maturation. This result is consistent with a previous finding that FGF23 concentration was elevated at birth and higher than reported in adults [36]. Moreover, the FGF23 is a circulating peptide produced by osteocytes. Previous studies have shown that there is a relationship between FGF23 and bone formation [37, 38], suggesting that the negative correlation between serum FGF23 level and age might be related to osteogenesis and skeletal maturation. However, the decreased serum FGF23 level with increasing age was only seen in younger children less than 3 years old. Data on 1,25-dihydroxyvitamin D and parathyroid hormone (PTH) levels were not available in the study, and thus the association between FGF23 and PTH could not be studied. Further studies are necessary to identify whether the association of serum FGF23 with age is in relation to osteogenesis and skeletal maturation.

Significant correlations between biomarkers and measures of kidney function assessed by eGFR were identified in the present study. Previous studies have suggested that eGFR based on both serum Cr and CysC levels is more accurate than equations based on either [34, 39]. Therefore, we calculated eGFR based on both serum Cr and CysC, and demonstrated that the association of eGFR with serum FGF23 levels persisted even after adjustment for age and body weight, indicating that serum FGF23 levels have an inverse relationship to kidney function. This result is in line with a previous study conducted in adult patients with preserved renal function, where higher plasma FGF23 concentration was associated with lower estimated GFR [40]. Our data highlight the need to determine whether serum FGF23 is a potential marker for monitoring kidney dysfunction in critically ill children in large multicenter studies.

To our knowledge, this study is the first to examine the relationships between serum and urinary IGFBP-7 and FGF23 levels with AKI in critically ill children. Of note, our observation of FGF23 levels in critically ill children with AKI is not consistent with previous research [16, 18, 19], and furthermore FGF23 levels in both urine and serum are not useful for the prediction of AKI in critically ill children. The most likely explanation for this discrepancy between our data and previous data could be that we evaluated the predictive accuracy of FGF23 in a general and heterogeneous PICU population rather than in a specific clinical setting, such as in patients undergone cardiac surgery [16, 18, 19] or in randomly selected ICU patients [14, 15]. Given the heterogeneity and dynamic nature of AKI, the predictive performance is dependent strongly on the underlying conditions. The poor results derived from a mixed heterogeneous PICU might be related to the low specificity of FGF23 for AKI. Indeed, upregulation of FGF23 was reported in patients with hypertension, advanced diabetic nephropathy, and cardiovascular disease [41] or in patients with end stage liver disease [42]. Our data support the concept that the usefulness of biomarkers should be addressed differently for different clinical settings [7]. In addition, the level of FGF23 was substantially influenced by age and body weight, which might be considered as disadvantages in the clinical utility of FGF23 as an AKI biomarker in PICU population. The age did not remain significantly associated with severe AKI after adjustment for illness severity in the present study, suggesting that the positive correlation of age with AKI might be due to the higher prevalence of severe underlying diseases in older children, rather than due to a direct effect of age.

One of our major findings was a significant association of urinary IGFBP-7 with severe AKI in critically ill children, which is in line with the previous report from Aregger et al. [20], where urinary IGFBP-7 was identified by proteomics as an early prognostic marker of AKI severity. We verified the use of urinary IGFBP-7 and evaluated the impact of urinary IGFBP-7 on predicting severe AKI in a general PICU population, independent of the severity of illness. It is well accepted that a desirable biomarker should be characterized by a high accuracy and unaffected by potential confounders. The odds ratio for urinary IGFBP-7 to predict severe AKI occurrence remained significant after adjustment for body weight and severity of illness, as assessed by PRISM III score, demonstrating that urinary IGFBP-7 was independently associated with increased risk for severe AKI in critically ill children.

Our study provides the first evidence of a significant association of urinary IGFBP-7 with severe AKI in critically ill children; however, urinaryIGFBP-7 level is not superior to serum or urinary CysC in predicting severe AKI. Since multiple pathways are involved in the development and progression of AKI, a single biomarker may be unlikely to provide the required predictive accuracy in general PICU population, and a panel of biomarkers for accurately predicting AKI might be necessary. Nevertheless, despite the biological diversity, the combination of urinary IGFBP-7 and serum or urinary CysC did not substantially improve the prediction of severe AKI in critically ill children.

The ROC curve analysis in the present study showed that serum CysC appeared to play a greater role in predicting severe AKI, which is in agreement with previous studies where serum CysC has been reported to be associated with an increased risk of AKI in various pediatric cohorts [8, 9]. Notably, although two studies have shown that serum CysC is an early and accurate biomarker for AKI in general critically ill children [8, 9], we are the first to demonstrate that serum CysC was independently associated with AKI, even after adjustment for body weight and illness severity as assessed by PRISM III score. Our results strongly indicate that serum CysC could serve as an independent biomarker to predict severe AKI in critically ill children.

This present study has some limitations. Firstly, we utilized elevated serum Cr levels as a reference standard to define AKI. Although serum Cr remains a widely used marker for evaluating kidney function in PICU, its disadvantage has been well discussed and recognized. Secondly, although the use of urine output criteria for AKI diagnosis has not been well validated [43], it has been suggested that patients meeting both serum Cr and urine output criteria for AKI have worse outcomes compared with patients who manifest AKI predominantly by one criterion [44]. The diagnosis and staging of AKI based only on serum Cr without urine output criteria may have under estimated incidence and grade of AKI. Thirdly, previous studies have indicated that AKI incidence is best estimated by choosing the lowest Cr value within the first week in the ICU as baseline Cr, suggesting that any reasonable estimate based on Cr measures is likely to be better than an estimate that takes into account only age, gender, and race [32]. However, the use of the lowest Cr value during hospitalization as the baseline Cr for patients with elevated serum Cr (≥106.1 μmol/L) at PICU admission has not been validated in critically ill children. Fourthly, the lack of serial measurements of these biomarkers during PICU stay might reduce the likelihood of observing the difference between AKI and non-AKI groups. Fifthly, although the urinary levels of IGFBP-7 and CysC were affected by sepsis; urinary IGFBP-7 and CysC were independently associated with increased risk for severe AKI, even after adjustment for the presence of sepsis. The present study was not powered to specifically detect differences in these biomarkers between septic children with versus without AKI. Finally, the relatively small sample size limited the power to perform logistic regression between these biomarkers and mortality.

## Conclusions

Our results have shown that serum FGF23 levels are inversely related to measures of eGFR, irrespective of illness severity, suggesting that the elevated serum FGF23 level may reflect a decline in kidney function independently. In contrast to serum and urinary FGF23 which are not associated with AKI in a general and heterogeneous PICU population, an increased urinary level of IGFBP-7 was independently associated with increased risk of severe AKI diagnosed within the next 5 days after sampling. However, urinary IGFBP-7 was not superior to serum or urinary CysC in predicting severe AKI in critically ill children. Further investigation is needed to explore the role of FGF23 and IGFBP-7 for prediction of AKI in various pediatric cohorts.

## Abbreviations

AKI: Acute kidney injury
AKIN: AKI network
AOR: Adjusted odds ratio
CI: Confidence interval
Cr: Creatinine
CysC: Cystatin C
eGFR: Estimated glomerular filtration rate
FGF23: Fibroblast growth factor 23
IGFBP-7: Insulin-like growth factor binding protein 7
IQR: Interquartile range
LOS: Length of stay
MV: Mechanical ventilation
OR: Odds ratio
PICU: Pediatric intensive care unit
PRISM III score: Pediatric risk of mortality III
PTH: Parathyroid hormone
